# INSIGHT responsive parenting intervention reduces infant’s screen time and television exposure

**DOI:** 10.1186/s12966-018-0657-5

**Published:** 2018-03-15

**Authors:** Elizabeth L. Adams, Michele E. Marini, Jennifer Stokes, Leann L. Birch, Ian M. Paul, Jennifer S. Savage

**Affiliations:** 1 Center for Childhood Obesity Research, Penn State University, 129 Noll Laboratory, University Park, PA 16802 USA; 2 Department of Nutritional Sciences, Penn State University, University Park, PA USA; 3 Department of Foods and Nutrition, University of Georgia, Athens, GA USA; 4 Pediatrics and Public Health Sciences, Penn State College of Medicine, Hershey, PA USA

**Keywords:** Obesity prevention, Media use in children, Infancy, Tummy time, Physical activity

## Abstract

**Background:**

Sedentary behaviors, including screen time, in childhood have been associated with an increased risk for overweight. Beginning in infancy, we sought to reduce screen time and television exposure and increase time spent in interactive play as one component of a responsive parenting (RP) intervention designed for obesity prevention.

**Methods:**

The Intervention Nurses Start Infants Growing on Healthy Trajectories (INSIGHT) study is a randomized trial comparing a RP intervention with a safety control intervention. Primiparous mother-newborn dyads (*N* = 279) were randomized after childbirth. Research nurses delivered intervention content at infant ages 3, 16, 28, and 40 weeks and research center visits at 1 and 2 years. As one component of INSIGHT, developmentally appropriate messages on minimizing screen time, reducing television exposure in the home, and promoting parent-child engagement through interactive play were delivered. Mothers self-reported their infant’s screen time at ages 44 weeks, 1, 1.5, 2 and 2.5 years; interactive play was reported at 8 and 20 weeks and 2 years.

**Results:**

More RP than control parents reported their infants met the American Academy of Pediatrics’ no screen time recommendation at 44 weeks (53.0% vs. 30.2%) and at 1 year on weekdays (42.5% vs. 27.6%) and weekends (45.5% vs. 26.8%), but not after age 1 year. RP mothers and RP children had less daily screen time than controls at each time point (*p* ≤ 0.01). Fewer RP than control group mothers reported the television was ever on during infant meals (*p* < 0.05). The frequency of tummy time and floor play did not differ by study group; approximately 95% of infants spent time in restrictive devices (i.e. swing) at 8 and 20 weeks. At 2 years of age, there were no study group differences for time children spent in interactive play.

**Conclusion:**

From infancy to early childhood, the INSIGHT RP intervention reduced screen time and television exposure, but did not increase the frequency or amount of interactive play.

**Trial registration:**

clinicaltrials.gov NCT01167270. Registered on 21 July 2010.

**Electronic supplementary material:**

The online version of this article (10.1186/s12966-018-0657-5) contains supplementary material, which is available to authorized users.

## Background

As part of routine care, health care providers deliver anticipatory guidance to parents of infants and young children, which include proactive recommendations to support healthy, emerging behaviors for appropriate growth and development. Such advice begins early in life and includes the avoidance of sedentary behaviors (e.g. television viewing) and the encouragement of physical activity. For example, in the United States, recommendations for “tummy time” occur at the 2-month well child visit [1] and the avoidance of television viewing and other screens (i.e. web-based programming, mobile/interactive technologies) is emphasized during infancy and early childhood [2, 3]. This early life guidance is intended to promote healthy long-term behaviors, given the numerous negative health consequences associated with screen time and sedentary behaviors [4–12], as well as the many positive outcomes associated with being physically active [13–15]. For example, screen time is associated with an increased body mass index (BMI) [4, 5], reduced sleep [6, 7], social/emotional delays [8, 9], decreased parent-child interactions, and less joint active play [10–12]. Parent-child play is particularly important during early childhood, as this cultivates learning, fosters creativity, and encourages parent-child relationships that promote healthy development [16].

Limited research has quantified infant screen time and interactive play (i.e. interactions that aid in physical and social development), particularly longitudinal patterns from infancy to early childhood. In a systematic review of children less than 2 years of age, average daily television exposure was widely variable (54.6–330.9 min per day) [17]. To reduce screen time and promote interactive play, the American Academy of Pediatrics (AAP) recommends 1) limited screen time for children < 2 years of age, 2) no screen time during meals, 3) no television in children’s bedroom, 4) daily interactive play for children, ideally with parent engagement, and 5) limited time that infants are in restrictive devices (e.g. swings, bouncers) [2, 3]. Yet, little evidence has quantified parents and children’s compliance with this anticipatory guidance provided by the AAP [1]. Further, this anticipatory guidance may not be common internationally; therefore, interventions can be designed to supplement this guidance and further promote associated, positive health outcomes. The Healthy Beginnings Trial [18], Prevention of Overweight in Infancy [19], and Infant Feeding Activity and Nutrition Trial [20] are among the few interventions that have targeted secondary outcomes of increasing interactive play and reducing sedentary behavior during infancy. These interventions were delivered across infants first 1.5 [19, 20] to 2 [18] years of life by nurse home visits [18] or group education sessions [19, 20] to first-time mothers [18, 20]. Both positive [21] and null [22] findings on increasing tummy time and/or physical activity resulted; therefore suggesting these as promising intervention targets that require additional research to increase effectiveness.

The Intervention Nurses Start Infants Growing on Healthy Trajectories (INSIGHT) trial is a responsive parenting (RP) intervention, designed to prevent rapid infant weight gain and overweight during early childhood [23]. RP guidance provided to mothers included responding promptly, contingently, and in developmentally appropriate ways during different states of infant arousal, including fussing/crying, active/alert, drowsy, and sleepy. As one component of INSIGHT, guidance based on AAP recommendations was provided to reduce screen time and increase parent-child time spent engaged in interactive play. We have previously shown that INSIGHT reduced rapid weight gain during the first 6 months of life [24], reduced the prevalence of overweight at age 1 year [24], increased sleep duration [25], and improved the dietary patterns of formula fed infants in the RP condition [26]. The aims of these analyses were to 1) describe mothers and children’s screen time, television exposure, and interactive play from infancy to early childhood and 2) examine the effects of INSIGHT on mother’s and children’s screen time, television exposure, and interactive play. We hypothesized 1) mother’s screen time would decrease and children’s screen time would increase from infancy to early childhood and 2) the RP group would have less screen time and television exposure, with greater time in interactive play, compared to the control group.

## Methods

### Participants and study design

Full details on the INSIGHT study design, eligibility criteria, and CONSORT diagram are published elsewhere [23, 24] (Additional file 1). Briefly, mothers were eligible for INSIGHT if they were primiparous, English-speaking, ≥20 years of age, and had a singleton infant born full-term (≥37 weeks gestation) with a birth weight ≥ 2500 g. Mothers were recruited from the maternity ward at a single center (Penn State Milton S. Hershey Medical Center, Hershey, PA, USA) from January 2012 to March 2014, then randomized 2 weeks after delivery into a responsive parenting (RP) or safety control group. Study groups were stratified by intended feeding mode (breast or formula) and birth weight for gestational age (<50th or ≥50th percentile). Trained research nurses delivered the INSIGHT RP and control intervention material to mothers during one-on-one home visits when infants were 3–4, 16, 28, and 40 weeks of age, and at a research center visit when infants were 1 and 2 years of age. Of the 316 participants enrolled, 291 were randomized, and a final cohort of *N* = 279 mother-infant dyads completed the first home visit. Participant retention was 83%, with *n* = 233 remaining in the study at 3 years. Mothers who dropped out of the study were more likely to be not married, Hispanic, Black, and younger than mothers who remained in the study. This study was approved by the Penn State College of Medicine’s Human Subjects Protection Office and is registered at clinicaltrials.gov.

### INSIGHT intervention curriculum

The INSIGHT curriculum uses a responsive parenting framework to teach parents to respond promptly, contingently, and in developmentally appropriate ways, with a focus on four infant behavioral states: drowsy, sleepy, fussy, and alert/calm. In the context of these four states, the RP group received messages around promoting child self-regulation, while the control group received messages on child safety [23]. INSIGHT’s central focus was to encourage responsive feeding to promote self-regulation of food intake and reduce overweight; yet, this multi-component intervention also included messages on parenting practices regarding sleep, emotional regulation, and interactive play. The intervention messages relevant to these analyses on screen time, television exposure, and interactive play are described in Table 1. The control group received child safety messages at the same time points that were matched for content intensity. These safety messages were centered on the same four infant behavioral states (drowsy, sleepy, fussy, and alert/calm) and avoided any messages that could impact energy balance. For example, fire safety, prevention of falls, and toy safety were discussed. The RP messages on screen time reflected the 2012 AAP guidelines, which were current at the time of intervention delivery [2].

**Table 1:** Timing of screen time, television exposure, and interactive play messages in the responsive parenting intervention curriculum

	Child age (weeks)				
Intervention messages	3–4	16	28	40	52
Screen Time and Television Exposure					
Limit screen time (i.e. televisions, computer)	X	X	X	X	X
No television in the child’s bedroom				X	X
No television on when infant is in the room		X	X		X
Never have the television on during meals				X	X
Interactive Play					
Practice tummy time several times per day	X	X	X		
Encourage on-the-floor free play		X	X	X	
Limit use of restrictive devices (i.e. swings)	X	X	X	X	
Interact and play with your infant	X	X	X	X	
Provide outdoor play time	X	X	X	X	
Model physical activity			X	X	

Screen time and television exposure were assessed at infant age 44 weeks, and 1, 1.5, 2, and 2.5 years. Interactive play was assessed at child age 2 years. Intervention curriculum not listed above included messages on feeding, sleep, and temperament

### Measures

Online surveys using REDCap [27] were used to collect and manage participant data. For those without Internet access, paper surveys were mailed to their home address (*n* = 20). Demographic information, such as race/ethnicity, income, and marital status, were collected at enrollment. Maternal age, pre-pregnancy weight, gestational weight gain, infant gestational age, and sex were abstracted from medical records. Infant feeding mode at 16 weeks of age was reported by mothers and defined as predominantly breastfed when ≥80% of milk feedings were breast milk, either at the breast or by bottle [28, 29].

### Screen time and television exposure

Mother’s and children’s screen time were assessed at child age 44 weeks, 1.5, and 2.5 years using an internally developed questionnaire, informed by previously validated questionnaires for parents of older children [30, 31]. Mothers were asked, “At home, how many hours a day do you/your child spend on screen time (includes television, video, iPad, computer and gaming systems)?” At 1 and 2 years of age, children’s screen time was also reported, but separately for weekdays and weekends. Children’s television exposure was assessed at 44 weeks, 1.5, and 2.5 years with items such as, “How many hours per day is the television typically on when you are at home?” “At home, how often does your child eat a meal while watching television?” and “At home, how often is the television on during snack times?” with response options of Never, Rarely, Sometimes, Usually, and Always. The number and location of televisions in the home were also asked.

### Interactive play questionnaire

Given the lack of available, published questionnaires developed for infants, questions to assess the frequency of interactive play were developed by lead study investigators. These items were designed to measure the frequency of developmentally appropriate playtime behaviors among infant/child-parent dyads. When infants were 8 and 20 weeks of age, mothers were asked, “How often does your baby spend time on his/her tummy when they are awake?” “How often do you play on the floor with your baby?” “Does your baby enjoy being on his/her tummy?” and “When does your baby usually spend time in a swing, bouncer, or other infant seat?”

The Physical Activity questionnaire [32] was used to assess the frequency and level of active play in children at age 2 years. This questionnaire was slightly modified to fit our study population (e.g. time reference of “in a typical week” changed to “in the past week”). Response options for the frequency of children’s daily active play on weekdays and weekends included 0, < 1, 1–2, 2–3, 3–4, and ≥4 h/day. Additional items developed by lead study investigators included, “When at home in the past week, how often did your child play outside?” and “In the past week, how often did you play outside or do active play inside with your child?” with response options of Never, Once, Sometimes, Almost Daily, and Daily.

### Statistical analysis

Repeated measures analysis of variance (RMANOVA) with a Tukey post hoc correction were used to examine longitudinal patterns of daily screen time and television exposure as affected by intervention group. For these models, main effects included study group and age (i.e, time). An age X study group interaction was included to test if changes over time differed by study group, and when not significant, the interaction term was removed from the model. Given previous literature on correlates with screen time [33–35], all RMANOVA models were adjusted for the following covariates: marital status, maternal age at recruitment, pre-pregnancy BMI, and income. Chi-square tests of independence were used to examine categorical outcome variables, with a Mantel-Haenszel correction used for longitudinal analyses. All analyses were performed in SAS Version 9.4 (SAS Institute Inc., Cary, NC), with statistical significance defined a priori as *p* < 0.05. Values are presented as mean ± standard error, with 95% confidence intervals showing the magnitude of differences where appropriate.

## Results

Participants were predominantly White, married, and college educated with about 80% reporting an annual household income ≥$50,000 (Table 2). At baseline, there were no study group differences on demographic variables.

**Table 2:** Participant demographics by study group (*n* = 279)

	Responsive Parenting (*n* = 140)	Control (*n* = 139)
Maternal demographics		
Age (years), mean (SD)	28.7 (4.6)	28.7 (4.9)
Pre-pregnancy BMI (kg/m^2^), mean (SD)	25.5 (5.0)	25.3 (5.6)
Gestational weight gain (kg), mean (SD)	15.6 (6.4)	15.0 (6.0)
Hispanic/Latino, n (%)	12 (8.6)	7 (5.0)
Race, n (%)		
Black	10 (7.1)	7 (5.0)
White	122 (87.1)	127 (91.4)
Native Hawaiian/Pacific Islander	1 (0.7)	0 (0)
Asian	5 (3.6)	4 (2.9)
Other (multi-racial)	2 (1.4)	1 (0.7)
Education, n (%)		
High school or less	16 (11.4)	16 (11.5)
Some college	37 (26.4)	36 (25.9)
College graduate	48 (34.3)	52 (37.4)
Graduate degree +	39 (27.9)	35 (25.2)
Married, n (%)	102 (72.9)	108 (77.7)
Annual household income, n (%)		
< $10,000	6 (4.3)	5 (3.6)
$10,000–24,999	10 (7.1)	10 (7.2)
$25,000–49,999	5 (3.6)	23 (16.6)
$50,000–74,999	46 (32.9)	26 (18.7)
$75,000–99,999	32 (22.9)	23 (16.6)
≥ $100,000	32 (22.9)	43 (30.9)
Do not know/refused to answer	9 (6.4)	9 (6.4)
Infant demographics		
Male sex, n (%)	75 (53.6)	69 (49.6)
Gestational age (weeks), mean (SD)	39.6 (1.2)	39.5 (1.1)
Birth weight (kg), mean (SD)	3.4 (0.4)	3.5 (0.4)
Birth length (cm), mean (SD)	50.9 (2.4)	50.7 (4.5)

### Daily screen time from infancy to childhood

**Fig. 1:**
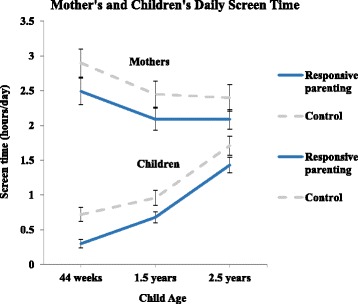
Longitudinal changes in average daily screen time for mothers and children. Values are mean ± SE. There were significant main effects of study group and child age on mother’s screen time (*p* < 0.01; *p* = 0.02, respectively) and on child screen time (*p* < 0.01; *p* < 0.01, respectively). There were no study group by age interactions for either mother or child’s screen time by child age (*p* ≥ 0.05)

There was no significant higher order age by study group interactions for daily screen time among mothers and children; therefore, main effects of age and study group are reported. Longitudinal patterns of daily screen time from infancy (age 44 weeks) to early childhood (age 2.5 years) indicated that, independent of study group, daily screen time increased with age (Fig. 1; *p* < 0.01). The average daily screen time at 44 weeks, 1.5 years, and 2.5 years was 0.7 ± 0.1, 1.0 ± 0.1, and 1.8 ± 0.1 h/day, respectively. Independent of age, children in the RP group had less daily screen time than children in the control group (Fig. 1; p < 0.01). In contrast, mother’s daily screen time from 44 weeks to 2.5 years postpartum decreased as children got older (Fig. 1; *p* = 0.02) independent of study group. Mother’s average daily screen time at 44 weeks, 1.5 years, and 2.5 years postpartum was 2.8 ± 0.1, 2.4 ± 0.1, and 2.4 ± 0.1 h/day, respectively. Mothers in the RP group reported less daily screen time than mothers in the control group (Fig. 1; *p* = 0.01), independent of maternal age.

More infants in the RP group than in the control group met the 2012 AAP guidelines for daily screen time duration at 44 weeks and at 1 year of age (*p* ≤ 0.02 for both), with no study group differences at 1.5, 2, and 2.5 years (Table 3). At 2 years of age, almost all children were meeting the AAP guidelines on weekdays (84.7%), with less meeting the AAP guidelines on weekends (71.6%).

**Table 3:** Proportion of children in the Responsive Parenting and Control group that met 2012 AAP screen time guidelines

	Met 2012 AAP guidelines (%)		
Child age	Responsive Parenting	Control	*p* value
44 weeks (*n* = 233)	53.0	30.2	< 0.01^*^
1 year			
Weekdays (*n* = 244)	42.5	27.6	0.02^*^
Weekends (*n* = 243)	45.5	26.8	< 0.01^*^
1.5 years (*n* = 228)	23.5	15.9	0.15
2 years			
Weekdays (*n* = 228)	87.8	81.4	0.18
Weekends (*n* = 229)	76.7	66.4	0.08
2.5 years (*n* = 212)	60.9	59.8	0.87

*AAP* American Academy of Pediatrics

At 44 weeks, 1 and 1.5 years, screen time guidelines were 0 h/day. At 2 and 2.5 years, guidelines were < 2 h/day. ^*^p < 0.05

### Television exposure in the home

There was no higher order age by study group interaction for television exposure in the home; therefore, main effects of age and study group are reported. The television was on in the home more hours per day, as children got older (44 weeks: 4.0 ± 0.2; 1.5 years: 6.4 ± 0.2; 2.5 years: 6.6 ± 0.2 h/day; *p* < 0.01). Independent of child age, the television was on in the home on average fewer hours per day for the RP group compared to the control group (5.4 ± 0.1 vs. 6.0 ± 0.1 h/day, respectively; *p* < 0.01). The median number of televisions in the home was 3 (range = 1 to 9), which did not differ by study group or across the study duration (*p* > 0.05). Few children had a television in their bedroom (age 44 weeks: 7.2%; 1 year: 7.4%; 2.5 years: 11.0%).

#### Characteristics associated with television exposure

We explored how marital status, maternal age at recruitment, pre-pregnancy BMI, income, and infant feeding mode were associated with hours per day the television was on in the home by study group. At infant age 44 weeks, for the control group, non-married mothers reported that the television was on in the home more hours per day than married mothers (5.8 ± 1.1 vs. 3.6 ± 0.2 h/day, respectively; *p* < 0.01); for the RP group, the television was on in the home a similar amount of hours per day for non-married and married mothers (3.6 ± 0.5 vs. 3.0 ± 0.2 h/day, respectively). The number of hours per day the television was on in the home was positively associated with pre-pregnancy BMI at 44 weeks postpartum and negatively associated with maternal age (*p* < 0.01) at 1.5 and 2.5 years postpartum (*p* = 0.01). Greater family income and predominantly breastfeeding were associated with less hours per day the television was on in the home at 44 weeks, 1.5, and 2.5 years postpartum (*p* < 0.03).

#### Television during meals and snacks

Fewer RP mothers, compared to control mothers, reported the television was ever on during infant meals at age 44 weeks (32.5% vs. 45.7%, respectively; *p* = 0.04), 1.5 years (48.7% vs. 68.1%, respectively; *p* < 0.01), and 2.5 years (66.4% vs. 78.4%; respectively, *p* = 0.05). The frequency of television on during children’s snacks did not differ by study group at any age.

### Description of interactive play

The INSIGHT RP intervention did not influence tummy time frequency at 8 and 20 weeks of age (Table 4). The majority of all infants met AAP recommendations for engaging in tummy time at least once per day (8 weeks: 80.9% met recommendations; 20 weeks: 86.2% met recommendations). More RP infants enjoyed tummy time “most of the time” at 8 weeks of age, compared to control infants (51.3% vs. 33.6%, respectively, *p* < 0.01), while at 20 weeks of age, the enjoyment of tummy time was similar across study groups. There were no study group differences in the frequency of mothers playing on the floor with their infant (Table 4). Approximately 95% of mothers reported their infant did spend time in swings, bouncers, or other infant seats. When asked what time of day their infant usually spent time in these devices, mothers most commonly reported the late afternoon (46.4%) or evening (49.4%) at 8 weeks and early morning (45.1%) or evening (51.2%) at 20 weeks.

**Table 4:** Frequency of mother-infant interactive play

	Responsive Parenting	Control	*P* value
Frequency of tummy time (%)			
8 weeks (*n* = 235)			0.25
Not every day, ≤6 days/week	13.0	25.0	
Every day, 1–2 times/day	44.4	45.0	
Every day, ≥3 times/day	42.6	30.0	
20 weeks (*n* = 246)			0.78
Not every day, ≤6 days/week	11.4	16.3	
Every day, 1–2 times/day	39.8	42.3	
Every day, ≥3 times/day	48.8	41.5	
Frequency of playing on the floor with infant (%)			
8 weeks (*n* = 223)			0.43
Less than 1 time/day	16.5	15.0	
1–3 times/day	63.5	60.0	
≥ 4 times/day	20.0	25.0	
20 weeks (*n* = 231)			0.69
Less than 1 time/day	3.3	7.4	
1–3 times/day	50.4	45.9	
≥ 4 times/day	46.3	46.7	

At 2 years of age, only 19.7% of children engaged in daily outdoor play when at home, with more RP children using an outdoor play area daily (30.0%), compared to children in the control group (15.1%) (*p* = 0.01). There were no study group differences in the frequency mothers and spouses/significant others engaged in physical activity with their child or in the frequency of children’s interactive play on weekdays and weekends. When dichotomized as < 4 h/day vs. ≥4 h/day, 56.3% and 62.8% of all children engaged in active play for ≥4 h/day on weekdays and weekends, respectively.

## Discussion

Infants in INSIGHT’s RP intervention were exposed to fewer hours of screen time and television exposure, resulting in more infants in the RP group meeting the 2012 AAP recommendations for screen time at 44 weeks and 1 year of age, compared to control infants. INSIGHT did not influence tummy time frequency, time spent in interactive play, or time spent in outdoor play at 2 years of age. Together with our results showing INSIGHT improved weight outcomes during the first year of life [24], dietary patterns [26], and infant sleep [25], our findings provide additional evidence on specific, modifiable RP strategies that can be used among caregivers of infants and young children to reduce screen-based activities during infancy.

Across all participants in INSIGHT, a greater percentage of mothers reported compliance with the AAP screen time recommendations when children were 2 and 2.5 years of age, compared to when children were < 2 years of age. Similar patterns are evidenced across a recent systematic review [17] showing low compliance among children 0–2 years of age for recommendations of no television viewing, while among children > 2 years of age [36], a greater percentage of parents reported compliance with the < 2 h/day screen time recommendations. Notably, INSIGHT increased the percentage of RP mothers who reported compliance with the AAP screen time recommendations during infancy, with no study group differences beyond age 1 year. This finding may be attributable to more frequent intervention visits in the first year of life with mothers receiving 4 nurse home visits and attending 1 research center visit. Only one visit occurred between ages 1 and 3 years, with limited guidance provided on screen time, television exposure, and interactive play. Our findings are promising in that INSIGHT increased compliance to the AAP guidelines during a period of life when compliance to these guidelines is typically low [6, 17, 22, 37, 38], while suggesting the need for frequent continuing guidance, particularly between ages 1 and 2 years. Pediatricians report the AAP recommendations on screen time are somewhat effective when delivered, with the most frequent barrier being a lack of parental motivation [39]. To augment the guidance delivered in well child visits and encourage parent engagement, personalized family media plans based on AAP recommendations can be used (https://www.healthychildren.org/English/media) [40].

INSIGHT reduced daily screen time for infants in the RP group, compared to those in the control group. Previous interventions aimed at reducing screen time in children have focused mainly on preschool and school-aged children [41–43], rather than infancy. Overall, these interventions have shown small but significant success in reducing screen time [42, 43], particularly among younger children [41]. Similar to the few early obesity prevention interventions that have begun during infancy [18–20], INSIGHT focused on the prevention of sedentary behaviors, rather than reducing already-established sedentary behaviors in later childhood. Further, the duration in which the television was on in the home (i.e, either being watched or as background noise) was lower for RP children (5.4 h/day) compared to control children (6 h/day). Reducing television exposure at a young age is important, due to its association with lower quality parent-child interactions [12] and reduced academic, psychosocial, and health behaviors in later childhood [44]. INSIGHT also reduced the reported television exposure during children’s meals for the RP group compared to the control group. Feeding young children with the television on is thought to decrease responsiveness during feeding, which may override children’s internal hunger and satiety cues [45]. For example, among preschoolers who frequently watched television, their energy intake during a single meal [46] and overall intake of discretionary foods high in saturated fat and/or added sugars was greater while television viewing [47]. Educating parents on the importance of reducing television exposure at a young age can establish healthy habits early in life, with the potential for enduring benefits that prevent the development of unhealthy eating behaviors later in life.

In our sample, greater maternal pre-pregnancy BMI, lower maternal age and family income, and not predominantly breastfeeding were associated with greater television exposure in the home. In a more diverse population, (e.g. minorities, non-married individuals, those experiencing depression), television exposure may be even greater [33, 35] with differences in television content [37]. For example, in a sample of predominantly non-married, low-income African American mothers, Thompson et al. [35] found infants were placed in front of a television for an average of 2.6 h per day at 3 months of age, with greater infant television exposure associated with greater maternal television viewing and obesity [35]. Higher socioeconomic index is also associated with greater child-directed programming, as opposed to adult-directed programming, during infant television viewing [37]. Lastly, among families enrolled in the Supplemental Nutrition Program for Women, Infants and Children, less parental viewing of electronic media and households with < 2 televisions had children more likely to meet the 2012 AAP guidelines on screen time [38]. Given these findings, interventions to reduce television exposure and screen time should consider tailoring intervention content for individuals with characteristics found to be associated with greater exposure. For example, family-centered interventions among low-income populations that aim to reduce parental media use and the number of televisions in the home may provide modifiable targets to limit screen time throughout infancy and childhood.

INSIGHT had less influence on interactive play behaviors. The majority of all infants engaged in daily tummy time (> 80%), daily mother-infant floor play (> 84%), time in movement restricting devices (~ 95%), and did not engage in daily outdoor play when at home at 2 years of age (~ 80%). These findings are similar to other interventions that have aimed to increase time spent in interactive play, specifically during infancy and young childhood [21, 48, 49]. One explanation for this may be the high percentage of infants (> 80%), regardless of study group, that engaged in daily tummy time, leaving little potential for improvement. Rather we suggest a greater emphasis on limiting time spent in movement-restricting devices and increasing outdoor play in future interventions, given the high percentage of children (≥80%), regardless of study group, that did *not* engage in this behavior. Perhaps the convenience and effectiveness of movement-restricting devices for soothing infants contributes to their frequent use, while parent’s time-constraints, work schedules, and seasonality may limit their ability to supervise young children’s outdoor play. To inform future interventions, qualitative research should explore parent’s perceived barriers for complying with the recommendations around restrictive devices and outdoor play during infancy/young childhood. Another possible explanation for the lack of study group differences for interactive play is that INSIGHT was not powered a priori to detect study group differences in these secondary outcomes.

To our knowledge, there are no valid and reliable questionnaires to quantify infant screen time, interactive play, or interactive play/screen time parenting practices; therefore, INSIGHT used existing literature to develop and modify existing measures in order to quantify these behaviors during infancy. We acknowledge the limitation of possible response bias in these self-reported data. RP mothers may have reported socially desirable behaviors that appeared compliant with the guidance they were provided, thus resulting in an overestimation of study group differences in our findings. To minimize this influence, mothers in this study completed the questionnaires independently and not in the presence of research staff; however, the development of objective measures for parent-child engagement in interactive play could be developed to further minimize response bias and improve the accuracy of characterizing these behaviors. In addition, the development of valid and reliable self-report questionnaires, specific to this population age, is needed for when objective measures are not feasible.

This INSIGHT trial did not collect information on the cost-effectiveness of the intervention. Given the importance of economic evaluation for scalability and dissemination of interventions, future work with INSIGHT will include analyses on its cost-effectiveness. Further, the screen time recommendations provided to RP mothers were based on the 2012 AAP guidelines, which were current at the time of intervention delivery [2]. In 2016 the AAP updated their screen time recommendations for young children to 1) discourage screen media, other than video-chatting, for children < 18 months of age, 2) choose high-quality media viewed with parents for children 18–24 months of age, and 3) limit high-quality media to 1 h per day for children 2–5 years of age [3]. We explored compliance to the 2016 AAP guidelines in our sample at age 2 and 2.5 years. As expected, a lower percentage of children would have met the 2016 guidelines of ≤1 h per day (34.5% - 49.1% at age 2; 15.7% - 18.2% at age 2.5) than children who met the 2012 guidelines of < 2 h per day (Table 3). More broadly, fewer children are likely meeting the 2016 screen time guidelines, as opposed to children who met the 2012 guidelines. Future research is needed to quantify compliance to the 2016 AAP guidelines after delivery of these specific recommendations to parents. Children’s use of different types of screen devices (e.g. video-chatting, high versus low-quality media) with and without parents present should also be quantified and compared to the 2016 AAP screen time guidelines.

## Conclusion

Previous research has revealed that RP can promote self-regulatory skills, socio-emotional, and cognitive development in children; findings from this study support the efficacy of INSIGHT RP strategies to reduce children’s screen time and television exposure beginning in infancy. Few other interventions have aimed to reduce screen time in young children [50]; INSIGHT is unique in that it began during infancy and continued through the first 2.5 years of life. The delivery of this anticipatory guidance on modifiable behaviors related to screen time and television exposure was effective during a critical period of infant’s development; yet, continued, more intensive anticipatory guidance beyond the first few years of life that also targets parent screen use may be needed to see persistent effects into childhood.

## Additional file

Additional file 1: INSIGHT study CONSORT diagram. Flow diagram detailing the number of participants who were screened, enrolled, randomized, and completed study visits up to infant age 1 year. (PDF 234 kb)

## Abbreviations

AAP: American Academy of Pediatrics
BMI: Body mass index
INSIGHT: Intervention Nurses Start Infants Growing on Healthy Trajectories
RMANOVA: Repeated measures analysis of variance
RP: Responsive parenting

## References

[1] Hagan JF, Shaw JS, Duncan PM (2017). Bright futures: guidelines for health supervision of infants, children and adolescents.

[2] Brown A (2011). Council on communications and media. Media use by children younger than 2 years. Pediatr..

[3] Council on Communications and Media (2016). Media and young minds. Pediatr.

[4] Cox R, Skouteris H, Rutherford L, Fuller-Tyszkiewicz M, Dell’ Aquila D, Hardy LL (2012). Television viewing, television content, food intake, physical activity and body mass index: a cross-sectional study of preschool children aged 2-6 years. Health Promot J Austr.

[5] Wen LM, Baur LA, Rissel C, Xu H, Simpson JM (2014). Correlates of body mass index and overweight and obesity of children aged 2 years: findings from the healthy beginnings trial. Obesity (Silver Spring).

[6] Cespedes EM, Gillman MW, Kleinman K, Rifas-Shiman SL, Redline S, Taveras EM. Television viewing, bedroom television, and sleep duration from infancy to mid-childhood. Pediatrics. 2014;133(5):e1163-71. 10.1542/peds.2013-3998 PMC4006444 24733878

[7] Vijakkhana N, Wilaisakditipakorn T, Ruedeekhajorn K, Pruksananonda C, Chonchaiya W (2015). Evening media exposure reduces night-time sleep. Acta Paediatr.

[8] Hinkley T, Verbestel V, Ahrens W, IDEFICS Consortium (2014). Early childhood electronic media use as a predictor of poorer well-being: a prospective cohort study. JAMA Pediatr.

[9] Pagani LS, Fitzpatrick C, Barnett TA, Dubow E (2010). Prospective associations between early childhood television exposure and academic, psychosocial, and physical well-being by middle childhood. Arch Pediatr Adolesc Med..

[10] Christakis DA, Gilkerson J, Richards JA (2009). Audible television and decreased adult words, infant vocalizations, and conversational turns: a population-based study. Arch Pediatr Adolesc Med.

[11] Schmidt ME, Pempek TA, Kirkorian HL, Lund AF, Anderson DR (2008). The effects of background television on the toy play behavior of very young children. Child Dev.

[12] Kirkorian HL, Pempek TA, Murphy LA, Schmidt ME, Anderson DR (2009). The impact of background television on parent-child interaction. Child Dev.

[13] Telama R, Yang X, Viikari J, Valimaki I, Wanne O, Raitakari O (2005). Physical activity from childhood to adulthood: a 21-year tracking study. Am J Prev Med.

[14] Telama R, Yang X, Leskinen E (2014). Tracking of physical activity from early childhood through youth into adulthood. Med Sci Sport Exerc.

[15] Reiner M, Niermann C, Jekauc D, Woll A (2013). Long-term health benefits of physical activity – a systematic review of longitudinal studies. BMC Public Health.

[16] Ginsburg KR, Committee on Communications, Committee on Psychosocial aspects of child and family health (2007). The importance of play in promoting healthy child development and maintaining strong parent-child bonds. Pediatrics.

[17] Downing KL, Hnatiuk J, Hesketh KD (2015). Prevalence of sedentary behavior in children under 2 year: a systematic review. Prev Med.

[18] Wen LM, Baur LA, Rissel C, Wardle K, Alperstein G, Simpson JM. Early intervention of multiple home visits to prevent childhood obesity in a disadvantaged population: a home-based randomised controlled trial (Healthy Beginnings Trial). BMC Public Health. 2007;7:76. 10.1186/1471-2458-7-76 PMC1877802 17490492

[19] Taylor BJ, Heath ALM, Galland BC, Gray AR, Lawrence JA, Sayers RM (2011). Prevention of overweight in infancy (POI.Nz) study: a randomized controlled trial of sleep, food and activity interventions for preventing overweight from birth. BMC Public Health.

[20] Campbell K, Hesketh K, Crawford D, Salmon J, Ball K, McCallum Z (2008). The infant feeding activity and nutrition trial (INFANT) an early intervention to prevent childhood obesity: cluster-randomized controlled trial. BMC Public Health.

[21] Wen LM, Baur LA, Simpson JM, Rissel C, Flood VM. Effectiveness of an early intervention for infant feeding practices and "tummy time": a randomized controlled trial. Arch Pediatr Adolesc Med. 2011;165(8):701-7. 10.1001/archpediatrics.2011.115 21810633

[22] Moir C, Meredith-Jones K, Taylor BJ, Gray A, Heath ALM, Dale K (2016). Early intervention to encourage physical activity in infants and toddlers: a randomized controlled trial. Med Sci Sports Exerc.

[23] Paul IM, Williams JS, Anzman-Frasca S (2014). The intervention nurses start infants growing on healthy trajectories (INSIGHT) study. BMC Pediatr.

[24] Savage JS, Birch LL, Marini M, Anzman-Frasca S, Paul IM (2016). Effect of the INSIGHT responsive parenting intervention on rapid infant weight gain and overweight status at age 1 year: a randomized clinical trial. JAMA Pediatr.

[25] Paul IM, Savage JS, Anzman-Frasca S, Marini ME, Mindell JA, Birch LL. INSIGHT responsive parenting intervention and infant sleep. Pediatrics. 2016;138(1):e20160762. 10.1542/peds.2016-0762 PMC4925087 27354460

[26] Hohman EE, Paul IM, Birch LL, Savage JS (2017). INSIGHT responsive parenting interventionis associated with healthier patterns of dietary exposures in infants. Obesity (Silver Spring).

[27] Harris PA, Taylor R, Thielke R, Payne J, Gonzalez N, Conde JG (2009). Research electronic data capture (REDCap)--a metadata-driven methodology and workflow process for providing translational research informatics support. J Biomed Inform.

[28] Li R, Fein SB, Grummer-Strawn LM (2008). Association of breastfeeding intensity and bottle-emptying behaviors at early infancy with infants’ risk for excess weight at late infancy. Pediatrics.

[29] Grummer-Strawn LM, Scanlon KS, Fein SB (2008). Infant feeding and feeding transitions during the first year of life. Pediatrics.

[30] Ihmels MA, Welk GJ, Eisenmann JC, Nusser SM (2009). Development and preliminary validation of a family nutrition and physical activity (FNPA) screening tool. Int J Behav Nutr Phys Act.

[31] Boutelle KN, Birnbaum AS, Lytle LA, Murray DM, Story M (2003). Associations between perceived family meal environment and parent intake of fruit, vegetables, and fat. J Nutr Educ Behav.

[32] Trost SG, Sirard JR, Dowda M, Pfeiffer KA, Pate RR (2003). Physical activity in overweight and nonoverweight preschool children. Int J Obesity.

[33] Duch H, Fisher EM, Ensari I, Harington A (2013). Screen time use in children under 3 years: a systematic review of correlates. Int J Behav Nutr Phys Act.

[34] Chandra M, Jalaludin B, Woolfenden S (2016). Screen time of infants in Sydney, Australia: a birth cohort study. BMJ Open.

[35] Thompson AL, Adair LS, Bentley ME (2013). Maternal characteristics and perception of temperament associated with infant TV exposure. Pediatrics.

[36] Khalsa AS, Kharofa R, Ollberding NJ, Bishop L, Copeland KA (2017). Attainment of ‘5-2-1-0’ obesity recommendations in preschool-aged children. Prev Med Reports.

[37] Barr R, Danziger C, Hilliard M, Andolina C, Ruskis J (2010). Amount, content and context of infant media exposure: a parental questionnaire and diary analysis. Int J Early Years Educ.

[38] Asplund KM, Kair LR, Arain YH, Cervantes M, Oreskovic NM, Zuckerman KE (2015). Early childhood screen time and parental attitudes toward child television viewing in a low-income Latino population attending the special supplemental nutrition program for women, infants, and children. Child Obes.

[39] Gentile DA, Oberg C, Sherwood NE, Story M, Walsh DA, Hogan M, American Academy of Pediatrics (2004). Well-child visits in the video age: pediatricians and the American Academy of Pediatrics guidelines for children’s media use. Pediatrics.

[40] American Academy of Pediatrics. Family Media Plan. https://www.healthychildren.org/English/media. Accessed 17 Aug 2017.

[41] Wahi G, Parkin PC, Beyene J (2011). Effectiveness of interventions aimed at reducing screen time in children: a systematic review and meta-analysis of randomized controlled trials. Arch Pediatr Adolesc Med..

[42] Wu L, Sun S, He Y, Jiang B (2016). The effect of interventions targeting screen time reduction: a systematic review and meta-analysis. Medicine (Baltimore).

[43] Maniccia DM, Davison KK, Marshall SJ, Manganello JA, Dennison BA (2011). A meta-analysis of interventions that target children’s screen time for reduction. Pediatrics.

[44] Pagani LS, Fitzpatrick C, Barnett TA (2010). Prospective associations between early childhood television exposure and academic, psychosocial, and physical wellbeing by middle childhood. Arch Pediatr Adolesc Med.

[45] Golen RP, Ventura AK (2015). What are mothers doing while feeding their infants? Exploring the prevalence of maternal distraction during infant feeding interactions. Early Hum Dev.

[46] Francis LA, Birch LL. Does eating during television viewing affect preschool children's intake? J Am Diet Assoc. 2006;106(4):598-600. 10.1016/j.jada.2006.01.008 PMC2596580 16567158

[47] Batis C, Rodriguez-Ramirez S, Ariza AC, Rivera JA. Intakes of energy and discretionary food in Mexico are associated with the context of eating: mealtime, activity, and place. J Nutr. 2016;146(9):1907S-15S. 10.3945/jn.115.219857 27511934

[48] Gross RS, Mendelsohn AL, Yin HS, et al. Randomized controlled trial of an early childhood obesity prevention intervention: impacts on infant tummy time. Obesity (Silver Spring). 2017; 25(5):920-27. 10.1002/oby.21779 PMC5404992 28332324

[49] Xu H, Wen LM, Hardy LL, Rissel C. A 5-year longitudinal analysis of modifiable predictors for outdoor play and screen-time of 2- to 5-year-olds. Int J Behav Nutr Phys Act. 2016;13:96. 10.1186/s12966-016-0422-6 PMC5000406 27561357

[50] Schmidt ME, Haines J, O'Brien A, McDonald J, Price S, Sherry B, Taveras EM. Systematic review of effective strategies for reducing screen time among young children. Obesity. 2012;20(7):1338-54. 10.1038/oby.2011.348 22222926

