# Supplementary figures and images for: INSIGHT responsive parenting intervention reduces infant’s screen time and television exposure
Source: Int J Behav Nutr Phys Act. 2018 Mar 15;15:24. doi: 10.1186/s12966-018-0657-5 (PMC5855973; doi:10.1186/s12966-018-0657-5)

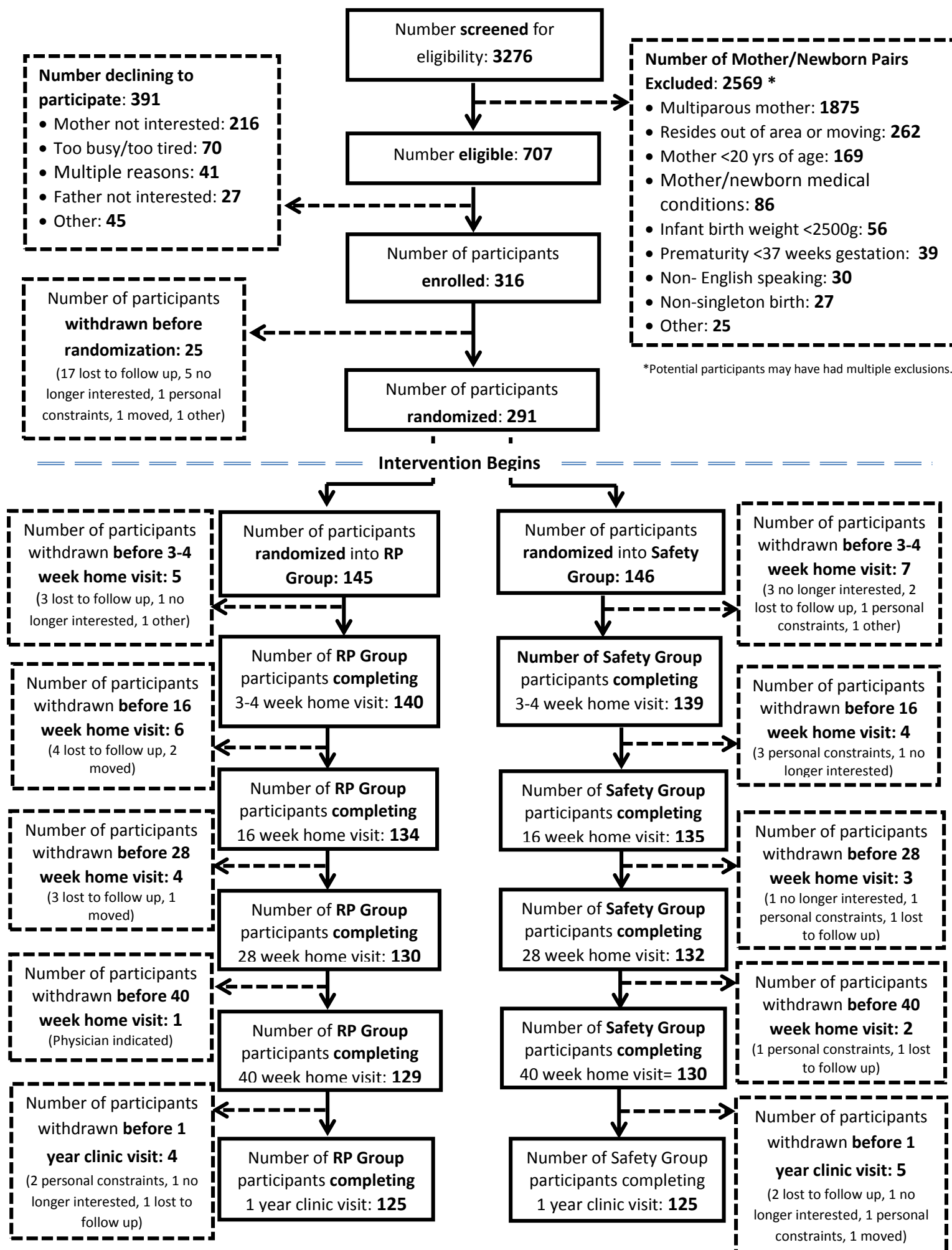

Supplement: Supplementary file 1 — INSIGHT study CONSORT diagram. Flow diagram detailing the number of participants who were screened, enrolled, randomized, and completed study visits up to infant age 1 year. (PDF 234 kb) [file 12966_2018_657_MOESM1_ESM.pdf]
